# Twenty years of fluorescence imaging of intracellular chloride

**DOI:** 10.3389/fncel.2014.00258

**Published:** 2014-08-29

**Authors:** Daniele Arosio, Gian Michele Ratto

**Affiliations:** ^1^Institute of Biophysics, National Research Council and Bruno Kessler FoundationTrento, Italy; ^2^Centre for Integrative Biology, University of TrentoTrento, Italy; ^3^Nanoscience Institute, National Research Council of ItalyPisa, Italy; ^4^NEST, Scuola Normale SuperiorePisa, Italy

**Keywords:** chloride imaging, inhibitory and excitatory neurotrasmission, Clomeleon, chloride binding site, intracellular chloride

## Abstract

Chloride homeostasis has a pivotal role in controlling neuronal excitability in the adult brain and during development. The intracellular concentration of chloride is regulated by the dynamic equilibrium between passive fluxes through membrane conductances and the active transport mediated by importers and exporters. In cortical neurons, chloride fluxes are coupled to network activity by the opening of the ionotropic GABA_A_ receptors that provides a direct link between the activity of interneurons and chloride fluxes. These molecular mechanisms are not evenly distributed and regulated over the neuron surface and this fact can lead to a compartmentalized control of the intracellular concentration of chloride. The inhibitory drive provided by the activity of the GABA_A_ receptors depends on the direction and strength of the associated currents, which are ultimately dictated by the gradient of chloride, the main charge carrier flowing through the GABA_A_ channel. Thus, the intracellular distribution of chloride determines the local strength of ionotropic inhibition and influences the interaction between converging excitation and inhibition. The importance of chloride regulation is also underlined by its involvement in several brain pathologies, including epilepsy and disorders of the autistic spectra. The full comprehension of the physiological meaning of GABAergic activity on neurons requires the measurement of the spatiotemporal dynamics of chloride fluxes across the membrane. Nowadays, there are several available tools for the task, and both synthetic and genetically encoded indicators have been successfully used for chloride imaging. Here, we will review the available sensors analyzing their properties and outlining desirable future developments.

## CHLORIDE REGULATION IN BRAIN CELLS

In the adult central nervous system, transient inhibitory activity is expressed by the synaptic release of GABA and glycine and by the activation of the relevant ionotropic receptors. The opening of the associated channels allows the flux of a current mainly carried by chloride ions moving along their electrochemical gradient and therefore, the intracellular concentration of chloride ([Cl]_i_) determines the amplitude of the inhibitory currents. The interest in measuring directly [Cl]_i_ increased with the dawning of the notion that GABA and glycine exert an excitatory action early during development due to the gradual evolution of the reversal potential of chloride (Obata et al., 1978; Ben-Ari et al., 1989; Cherubini et al., 1990; Wu et al., 1992; Kandler and Friauf, 1995; Ehrlich et al., 1999). This discovery suggested a complexity of the mechanisms underlying the regulation of [Cl]_i_ that was previously unsuspected (Ben-Ari, 2002). In all these studies, the development of chloride homeostasis was inferred by measuring the reversal potential of the GABA_A_ current, rather than by a directly measuring the concentration. This method continues to produce important insights in the field, but it has some drawbacks: (a) measurements of the reversal potential provide an imperfect estimate of intracellular chloride because the GABAergic current contains a bicarbonate component (see later); (b) [Cl]_i_ can be affected by the pipette content unless recording is performed in the perforated patch configuration which is characterized by an inherently larger and more variable access resistance; (c) measurements of the reversal potential of the GABAergic current *in vivo* would be extremely challenging and labor-intensive.

Regardless, these studies have paved the way to the present understanding of [Cl]_i_ regulation: an equilibrium between passive fluxes through membrane conductance, and energy-dependent fluxes mediated by the cotransporters NKCC1 and KCC2 (Delpire, 2000). Passive fluxes follow the chloride electrochemical gradient, while the cotransporters are responsible for moving chloride away from equilibrium. Both cotransporters are driven by ionic gradients, with NKCC1 using the sodium gradient to move potassium and chloride into the cell and KCC2 using the potassium gradient to move chloride out. In the immature brain, NKCC1 is the most abundant molecule determining high [Cl]_i_ levels. During normal brain development, however, NKCC1 expression decreases meanwhile KCC2 expression increases. These concomitant events decrease the [Cl]_i_ to a level consistent with its inhibitory role in the adult brain (Plotkin et al., 1997; Clayton et al., 1998; Lu et al., 1999; Rivera et al., 1999) – see also (Glykys et al., 2014) for an alternative view.

The activity of GABA_A_ currents has been estimated by indirect methods. For example, in the immature cortex, depolarizing GABA_A_ transmission can be unveiled by the opening of calcium voltage sensitive channels, which can be quantified by calcium imaging (Canepari et al., 2000; Ganguly et al., 2001). Also voltage sensitive dyes can be used to estimate GABAergic currents in slice preparations (Canepari et al., 2010).

The steady state concentration of intracellular chloride is given by the equilibrium between cotransporters, leakage, and tonic activation of GABA_A_ conductance. The time modulation of chloride concentration is caused by the incoming transient activation of synaptic GABA_A_, in cortical neurons, or glycine receptors, in the spinal cord, basal forebrain, and retina. Interestingly, both the GABA_A_ and the glycine ionotropic receptors are permeable to bicarbonate, which represents about 11 and 20% of the current flowing through the glycine receptor and GABA_A_ conductance respectively (Bormann et al., 1987; Kaila et al., 1993). Moreover, the flux of bicarbonate modulates intracellular pH establishing a coupling between chloride fluxes and pH change; a fact with important physiological consequences, but also directly affecting imaging, since all genetically encoded chloride sensors are sensitive to pH changes.

## WHY MEASURE INTRACELLULAR CHLORIDE IN BRAIN CELLS?

Since its onset, fluorescent imaging of ionic concentration had a long love affair with the neurosciences and it is easily argued that, in association with patch clamp, it has revolutionized our understanding of brain cell physiology. The key advantage of imaging is to allow the temporally and spatially resolved measurement of ionic concentration in brain cells in cultures, in acute or chronic slices and, by exploiting two photon excitation, also *in vivo*. The spatial resolution of these measures not only allows scientists to study different cells separately, but to image changes occurring in different sub cellular domains as minute as dendritic spines (Guthrie et al., 1991; Müller and Connor, 1991). Most imaging studies have been performed with sensors for calcium, because of this ion’s essential role in the regulation of intracellular signaling and biochemistry. Furthermore, the dynamics of [Ca]_i_ are considered as a proxy that reveals the underlying electrical activity of neurons: elevations in calcium are interpreted as evidence of net excitatory input and firing patterns can, at least in principle, be reconstructed from the time course of the somatic calcium changes (Smetters et al., 1999). Calcium increases are caused by the activation of voltage sensitive calcium channels that open in response to membrane depolarization. In the post-synaptic volume, calcium increases report both the activation of voltage sensitive channels and the incoming excitatory input though NMDA receptors (Yuste and Denk, 1995; Yuste et al., 1999; Noguchi et al., 2005). Thus, the spatially and temporally resolved imaging of calcium changes holds the key to decode the integration of excitatory synaptic inputs on a neuron. Just as calcium fluxes indicate neuronal firing and integration of excitatory inputs, chloride fluxes signal the activation of the ionotropic GABA currents, which are the main mediator of synaptic inhibition in the postnatal cortex. Therefore, imaging of chloride fluxes could well represent a crucial tool to understand the dynamic arrangement of ionotropic inhibitory inputs on neurons.

The interpretation of the measures of intracellular concentration must take into account a fundamental difference between the electrochemical state of equilibrium for calcium and chloride. The reversal potential of calcium is so positive that it is outside the range of potentials experienced by cells, such that the opening of a calcium conductance always results in calcium influx. Furthermore, given the strong buffering of calcium, neuronal firing leads to only relatively modest increases in free calcium concentration under normal physiological conditions, and thus the driving force for calcium is only marginally dependent on activity. The situation is different for chloride: because its gradient is rather shallow, the chloride reversal potential is not strongly negative and depolarizes further when [Cl]_i_ increases. If [Cl]_i_ exceeds about 20–25 mM, the opening of GABA_A_ channels depolarizes the membrane potential of a resting neuron toward the threshold potential for the activation of sodium channels. This dual role for GABA is often referred to as “hyperpolarizing” or “depolarizing” GABA (Ben-Ari, 2002). It is clear that the switch between these two drastically different operation modes for GABA_A_ action, depends critically on the absolute value of [Cl]_i_, providing a paradigmatic example of the importance of directly measuring chloride concentration. In early embryonic and postnatal life, GABA acts as the primary excitatory neurotransmitter. This occurs because chloride concentration is so high that the opening of GABA_A_ receptors brings neurons over the action potential threshold. As the glutamatergic transmission matures, [Cl]_i_ decreases, opening of GABA_A_ channels hyperpolarizes the neuron’s membrane potential and chloride fluxes are directed into neurons; henceforth GABA_A_ assumes its inhibitory role. The experimental evidences of this shift are extensive (see Ben-Ari, 2002 for a review) and, although imaging measurements of the developmental shift of [Cl]_i_ were obtained in neuronal cultures (Kuner and Augustine, 2000) and brain slices (Berglund et al., 2006; Glykys et al., 2009, 2014), a direct and pH independent measurement of chloride concentration *in vivo* during development has yet to be produced.

Astrocytes are the second great family of cells in the brain. In the last two decades we have learned that they do not simply play a crucial role in the control of the extracellular homeostasis and on the coupling between brain and circulation but they also exert important modulatory effects on synaptic plasticity (Perea et al., 2009; Araque et al., 2014). Although astrocytes do not possess a significant complement of voltage dependent conductances, they are endowed with a membrane that is enriched with a cohort of channels and transporters (Kirischuk et al., 2012); a fact that suggests a finely regulated intracellular environment. Indeed, in the last decade calcium imaging has revealed that astrocytes have a highly dynamic internal life, characterized by a complex spatiotemporal activity (Zorec et al., 2012). Astrocytes and neurons are engaged in a complex bidirectional dialog: synaptic activity modulates the internal state of astrocytes and, in turn, they release gliotransmitters that modulate neuronal function and synaptic transmission (Perea et al., 2009). Although astrocytes are not excitable in a classic sense, they interact with the extracellular and extrasynaptic environment through a multitude of mechanisms that are in many ways more complex than the ones operating in neurons. Ionic regulation in astrocytes is rather complex as underlined by the effects of neuronal activity on the intracellular concentration of calcium and sodium. Although the field has been little explored, the available biochemical evidences suggest that also chloride fluxes in astrocytes are coupled to neuronal activity. Chloride in astrocytes is regulated by the concerted activity of cotransporters and chloride channels. The cotransporters for GABA and glutamate are central to astrocyte function. Interestingly, the operation of glutamate and GABA transporters are coupled to fluxes of protons and chloride, respectively. Thus, we can expect to see changes in intracellular pH and chloride also in astrocytes during intense synaptic function and, hopefully, this will open a new window on the understanding of the activity dependent interplay between neurons and glia (Egawa et al., 2013).

## CHEMICAL INDICATORS

The earliest indicators for chloride imaging were synthetic dyes based on quinoline and they come in three variants: 6-methoxy-*N*-(3-sulfopropyl)quinolinium (SPQ), *N*-(ethoxycarbonylmethyl)-6-methoxyquinolinium bromide (MQAE) and 6-methoxy-*N*-ethylquinolinium iodide (MEQ). All these dyes have a similar mechanism of operation: an excited molecule by colliding with a chloride ion, returns to the fundamental state through a non-radioactive path. An increase in chloride concentration is thus signaled by a decrease in fluorescence without any change in either the excitation or emission spectra. This molecular process is commonly described as dynamic quenching of the sensor fluorescence. Chemical indicators are usually not ratiometric and thus cannot provide an absolute estimate of chloride concentration; in fact, their fluorescence intensity depends not only on chloride concentration but also on the dye concentration and optical thickness at each location. In principle it is possible to assemble a ratiometric dye by coupling the chloride sensitive molecule with a chloride insensitive dye. This has been done by Jayaraman et al. (1999), but to our knowledge, this sensor is not commercially available. On the positive side, these dyes are rather insensitive to bicarbonate concentration and pH variations, moreover, their kinetics for chloride association is very fast. For indicators based on fluorescence dynamic quenching, the Stern–Volmer equation describes the relationship between fluorescence and chloride concentration:

(1) $$F\,\left(\left[C\,l\right]\right)=\frac{F_{0}}{1+K_{S\,V}\,\left[C\,l\right]}$$

The constant *K_SV_* is the chloride concentration that quenches half of the fluorescence and *F_0_* is the fluorescence in zero chloride. The Stern–Volmer constant *K_SV_* is a key parameter which defines the optimal working range for each dye; it should be determined in the cells of interest since it can depend significantly on the chemical and physical characteristics of the cytosolic environment, e.g., temperature, ionic strength, and interaction with membrane structures. Quinolinium-based dyes have their excitation peak at about 350 nm and emission at 440–460 nm; this is unfortunate since UV excitation is strongly prone to cause photodamage and it has a very limited penetration in deep tissues. These chemical indicators suffer from further experimental limitations that have curtailed their usefulness: their loading and stable retention are suboptimal especially at temperatures above 30°C (Fukuda et al., 1998), and they are also prone to photobleaching (Inglefield and Schwartz-Bloom, 1997; Nakamura et al., 1997).

Notwithstanding these problems, important results have been obtained with these dyes even if the calibration procedures and controls necessary to overcome their limitations can be rather complex and provide only limited quantitative control on the measurement – see (Zhang et al., 2006) for an interesting analysis of MEQ signals. These early exploratory efforts at imaging the world of chloride distribution improved our understanding on both chloride regulation and the role of symporters in a variety of brain systems, including cortical neurons (Inglefield and Schwartz-Bloom, 1997), rod retinal cells (Thoreson et al., 2003) and motorneurons (Chub et al., 2006). These studies have also provided insights into pathological conditions that are affected by dysregulation of chloride homeostasis, a theme that is now in great development (White et al., 1997; Inglefield and Schwartz-Bloom, 1998; Sah and Schwartz-Bloom, 1999; Yamada et al., 2001). As a testimony of the ingenuity of the experimenters, chemical dyes have also provided an estimate of the changes occurring in the absolute chloride concentration during brain development, providing support to the depolarizing GABA model (Fukuda et al., 1998; Isomura et al., 2003; Zhang et al., 2006).

Most of these studies have employed conventional wide-field illumination, but chemical dyes can also be used with scanning microscopes. This application has lagged behind, likely because of the cost of the early UV lasers. The introduction of relatively low cost solid state blue and UV lasers might increase the potential usefulness of these dyes. In acute brain slices loaded with MEQ, UV laser-scanning confocal microscope revealed GABA-mediated changes of [Cl]_i_ in single neurons (Inglefield and Schwartz-Bloom, 1997). The authors showed that pressure injection of muscimol, an agonist of the GABA_A_ receptor, causes rapid chloride fluxes; demonstrating how chloride imaging can reveal transient inhibitory activity. More recently, an important step toward chloride imaging *in vivo* came with the demonstration that MQAE can be imaged by two photon excitation (Marandi et al., 2002; Kovalchuk and Garaschuk, 2012). Here, the membrane permeable dye MQAE has been successfully loaded in acute brain slices and it was found that two photon excitation causes much less photodamage and photobleaching than UV illumination. This property enables longer recordings and measurements of chloride fluxes in dendrites. A quite interesting fact derives from the analysis of the collisional quenching mechanism that is involved in the operation of these dyes: the higher the concentration of chloride ions, the shorter the average time elapsing between collisions. If the concentration is high enough that the mean interval between collisions is shorter than the mean lifetime of fluorescence, the observed lifetime of the dye is shortened with increasing concentration of chloride (Kaneko et al., 2002). The consequence of this is that the measurement of the fluorescence lifetime provides an absolute measure of chloride concentration that is free from the influence of sensor concentration. This property has been used: to measure the absolute concentration of chloride in the olfactory epithelium and demonstrate the existence of a chloride concentration gradient between the sensory dendrites and the cell body (Kaneko et al., 2004); to follow the maturation of chloride homeostasis in dorsal root ganglia (Gilbert et al., 2007); and to monitor the effects of inflammatory processes on chloride regulation (Funk et al., 2008).

## GENETICALLY ENCODED INDICATORS

Genetically encoded indicators are based on the green fluorescent protein (GFP) from the jellyfish *Aequorea victoria.* The potential of GFP as a molecular probe was first recognized when it was expressed heterologously in nematode to track gene expression in the sensory neurons (Chalfie et al., 1994). Remarkably, the chromophore of GFP is formed by an auto catalytic, post-translational cyclization and oxidation of the polypeptide backbone; making the GFP fluorescence genetically encodable. Since then, GFP has been extensively engineered to improve folding and maturation in eukaryotes at 37°C (Cubitt et al., 1995), to enhance fundamental fluorescence properties like brightness and photostability (Heim et al., 1995), and to expand the available color palette (Heim and Tsien, 1996). GFP and GFP homols, discovered in species other than *Aequorea victoria*, have become essential probes in cell biology (Rizzo et al., 2009) and appreciated in a wide variety of applications (Day and Davidson, 2009; Chudakov et al., 2010).

While the pH dependence of GFP fluorescence was already known, the opportunity to quantify [Cl]_i_ using GFP-based indicators arose when it was discovered that the fluorescence of the yellow fluorescent protein (YFP) – a GFP containing S65G, V68L, S72A, and T203Y mutations – strongly depends also on the environment concentration of halogens (Wachter and Remington, 1999). On the one hand, point mutations were introduced into YFP to reduce its sensitivity to pH and anion-concentration changes leading to two well known FP: Citrine (Griesbeck et al., 2001), which contains the Q69M mutation, and Venus (Nagai et al., 2002), which contains the F46L, F64L, M153T, V163A, and S175G mutations. On the other hand, few pioneering studies recognized in the halogen sensitivity of YFP fluorescence an opportunity for developing novel methods to measure intracellular anion concentration (Jayaraman et al., 2000). In particular, it was found that the introduction of the H148Q mutation into YFP produced a highly fluorescent protein with higher affinity for halides (**Table 1**). Crystallographic analyses showed that YFP-H148Q contains a specific binding site for halides (Wachter et al., 2000). YFP-H148Q was successfully used to develop a cell-based high-throughput assay for screening of agonists against cystic fibrosis transmembrane conductance regulator (CFTR)-mediated halide transport (Galietta et al., 2001b). In addition, YFP-H148Q was used as a starting template for generating random libraries of mutants that could have the potential for enhanced chloride affinity (Galietta et al., 2001a). The analysis of 1536 clones resulted in the discovery that in YFP-H148Q chloride affinity can be improved by the introduction of hydrophilic amino acid at position 150 and 163 (**Table 1**). A high-throughput screening based on the YFP-H148Q-I152L mutant was established for the screening of novel antagonists of GABA_A_, GABA_C_ and glycine receptors in HEK293 transfected cells (Kruger et al., 2005). The same YFP-H148Q-I152L mutant was also used for identifying inhibitors of a human calcium-activated chloride channel (De La Fuente et al., 2008), engineering a cell-based imaging of sodium-iodide symporter activity (Rhoden et al., 2007), and developing a multiplexed high-throughput flow cytometry analysis of the glycine receptor (Gilbert et al., 2009). Among the YFP-H148Q mutants, YFP-H148Q-V163S has been recently tagged at the N-terminus with a palmitoylation sequence and used for monitoring of intracellular chloride changes in neuronal processes by live-cell imaging of the midbrain (Watts et al., 2012). Membrane targeting of this mutant was shown not to alter the high sensitivity for chloride and to be advantageous for stabilizing the fluorophore during whole-cell patch-clamp recordings. As with all genetically encoded sensors, GFP-based chloride probes show key advantages over chemical probes: (1) they can be targeted conditionally to specific cell types and to specific sub cellular compartments; (2) they are retained within cells allowing chronic repeated measurements *in vivo;* (3) they are generally more photostable than chemical dyes; (4) in contrast to chemical dyes, the thermodynamic and kinetic parameters for chloride binding to GFP-based probes are concentration independent leading to more robust estimates of the calibration parameters. Dyes like MQAE, in fact, display large changes of the Stern–Volmer constant between calibration *in vitro*, *K_SV_*= 185 M^-1^, and *in vivo*, *K_SV_* from 2 to 40 M^-1^ (Kaneko et al., 2004). This large concentration-dependent variability is probably a result of self-quenching at high dye concentrations (>100 μM) and of dye interactions with other anions, e.g., HPO_4_^2-^ and HCO_3_^-^, within cells (Kaneko et al., 2004).

**Table 1:** Chloride and iodide affinity for various probes at pH 7.5 and room temperature.

Probe	Cl^-^ *K_d_* (mM)	I^-^ *K_d_* (mM)	Selected reference
MQAE N-(6-methoxyquinolyl) acetoethyl ester	13* ^in cuvette^		Marandi et al. (2002)
	40* ^in cells^		
SPQ 6-methoxy-*N*-(3-sulphopropyl) quinolinium	118*	276*	Krapf et al. (1988)
	8.5		
YFP	777		Wachter et al. (2000)
YFP-H148Q	197	20	Galietta et al. (2001a),
	154.4	23.2	Wachter et al. (2000)
YFP-H148Q-I152L	88	3	Galietta et al. (2001a)
YFP-H148Q-V150A-I152L	61	9	Galietta et al. (2001a)
YFP-H148Q-V163S	62	107	Galietta et al. (2001a)
YFP-H148Q-V163T-F165Y	55	5	Galietta et al. (2001a)
YFP-H148Q-V163L	77	21	Galietta et al. (2001a)

*Values for the Stern–Volmer constant are reported in place of the dissociation constant values.

## RATIOMETRIC CHLORIDE INDICATORS

Chloride indicators based on a single (Y)FP provide a single optical signal that cannot properly account for variations in local probe concentration, optical path length, light scattering, illumination intensity, and photobleaching. It has been repeatedly proven that these difficulties can be overcome by measuring the ratio between two optical signals that possess different dependency on the physiological parameter under study. Depending on the spectral properties of the sensor, these signals can be obtained by measuring two fluorescence emission bands or by changing the excitation wavelengths. Moreover, ratiometric methods allow accurate and precise measurement even in cells with complicated geometry such as neurons (Bullen and Saggau, 1999).

**FIGURE 1:**
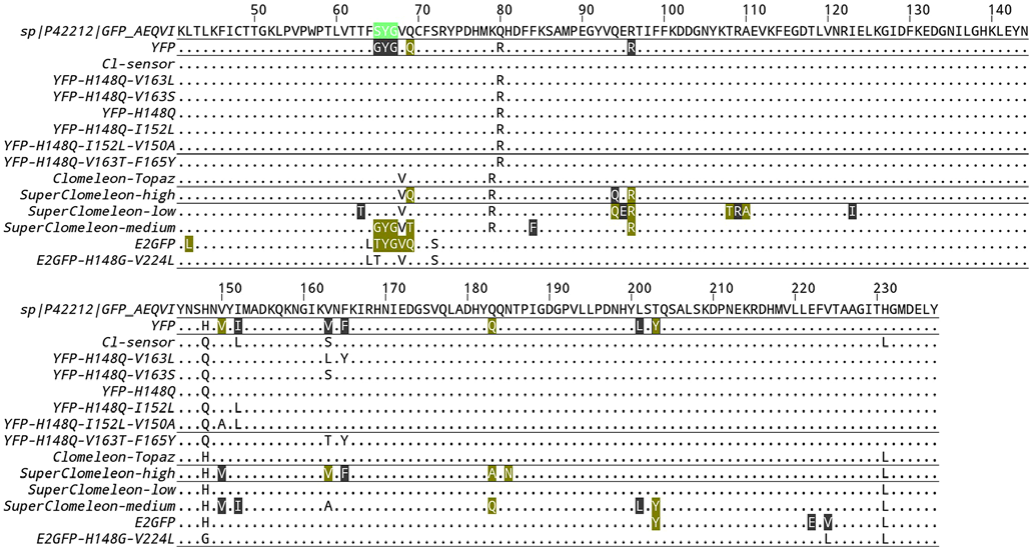
**Alignment of chloride binding GFP variants.** Only residues that differ from the reference wild-type GFP sequence (UniProtKB entry P42212) are indicated. If the protein data bank contains structural information of a given GFP variant, the residues of the chloride binding site are boxed. Color highlight indicates a residue that forms either van-der-Walls or hydrogen bonds to the chloride ion. Gray highlight indicates a residue that is located within a radius of 5 Å around the chloride ion without forming any specific type of bond.

Indeed, a crucial development in genetically encoded chloride biosensors occurred with the development of the first ratiometric YFP-based chloride biosensor Clomeleon (Kuner and Augustine, 2000). Clomeleon consists of two fluorescent proteins, CFP and Topaz, joined by a flexible polypeptide linker of 24 amino acids. Topaz is a YFP mutant, containing the H79R and L68V mutations (see alignment in **Figure 1**), which is sensitive to chloride ions. Because of the spectral overlap between CFP emission and Topaz absorbance, and the close spatial proximity between the two fluorophores, fluorescence resonance energy transfer (FRET) occurs. Chloride binding to Topaz reduces its emission, and leads to a decrease in the degree of FRET or, in more simple words, a change in color from yellow to cyan of the emitted fluorescence. As the CFP fluorescence is free from the influence of chloride concentration, exciting the CFP (at 440 nm) and measuring the emission ratio of Topaz relative to CFP (485 ± 15 nm/530 ± 15 nm) provides an absolute measurement of the chloride concentration. Clomeleon allowed studies to image synaptic inhibition and [Cl]_i_ in cultured neurons; and it has since been used in a wide range of applications both in slices and in culture. A line of transgenic mice that express Clomeleon under the control of the neuron-specific thy1 promoter is available (Berglund et al., 2006) and these mice have been used to demonstrate the spatiotemporal dynamics of inhibitory activity in various brain areas, from hippocampus to deep cerebellar nuclei (Berglund et al., 2008). Expressed in *Arabidopsis*, Clomeleon allowed chloride imaging in plants (Lorenzen et al., 2004). Clomeleon-based imaging was also demonstrated in a wide range of microscopy setup and techniques, including fluorescence lifetime imaging (Jose et al., 2007), 2-photon excitation microscopy (Berglund et al., 2006; Duebel et al., 2006; Dzhala et al., 2012; Glykys et al., 2014) and confocal spinning-disk (Berglund et al., 2006).

Clomeleon is unfortunately characterized by a few but significant limitations. Its signal displays substantial and complicated pH-dependence, and its chloride affinity is far from physiological [Cl]_i_. Chloride dissociation constant values from 87 to 167 mM were reported (Kuner and Augustine, 2000; Berglund et al., 2006; Dzhala et al., 2012) whereas physiological [Cl]_i_ lies within the range from 3 to 60 mM. The large variability of the reported values for chloride *K_d_* could be explained by the steep dependence of the chloride *K_d_* on pH. In fact the apparent chloride *K_d_* of Clomeleon spans two orders of magnitude in the pH range from 6 to 8 (Bregestovski and Arosio, 2011). While it is well known that fluorescence emission intensity is substantially affected by pH variations, it is less recognized that pH variations can alter the degree of FRET reported by numerous FRET-based biosensors. In general, pH changes around physiological values influence both the acceptor and the donor fluorescences altering their excitation and emission spectra as well as their Forster distance, at which the efficiency of FRET is 50%. Assuming that the average donor-acceptor distance is predetermined by the design of the biosensor construct, a pH variation itself can modify the FRET efficiency of any GFP-derived donor-acceptor pair. In Clomeleon, pH changes perturb the FRET-based chloride measurement in a manner that is difficult to predict and correct. In particular, pH-dependent changes of (donor) CFP lifetime have been analyzed in a detailed study that successfully exploited Clomeleon in fluorescence lifetime imaging microscopy despite the complex multiexponential pH-dependent fluorescence decay dynamics of the Clomeleon CFP donor (Jose et al., 2007).

For all these reasons, the development of ratiometric chloride biosensors with higher affinity for chloride and reduced pH-dependence was highly sought after. The first improvement on Clomeleon that enhanced the affinity for chloride was achieved by Cl-sensor (Markova et al., 2008), in which the chloride sensing fluorescent protein Topaz was substituted by the YFP-H148Q-I152L-V163S mutant. The YFP-H148Q-I152L-V163S mutant combines two mutations that were previously identified for their higher chloride affinities relative to YFP-H148Q (**Table 1**). As in Clomeleon, YFP-H148Q-I152L-V163S is joined to CFP by a flexible 20-amino acid linker. Cl-sensor calibration in cells yielded a remarkable value of 30 mM for the chloride *K_d_*, a value that matches the physiological [Cl]_i_ optimally. The excitation spectra of Cl-sensor is characterized by the presence of a Cl-independent isosbestic point at 465 nm, which is a useful feature for the recordings of chloride transients using conventional microscopes and rapid switching between excitation wavelengths. Borrowing the same design of Clomeleon, Cl-sensor displays the same subtle pH-dependence of the FRET readout, which hinders precise and pH-independent chloride measurements. Recently, a line of transgenic mice expressing Cl-sensor under the Thy1 promoter has been generated (Batti et al., 2013).

The optimization of Clomeleon-based biosensors is a process not yet completed. By cell-free protein engineering methods, a random library of Clomeleon variants was recently synthesized and screened for improved chloride affinity and dynamic range (Grimley et al., 2013). This synthetic approach, which bypasses cloning steps, was used to investigate how the performance of Clomeleon is influenced by seven residues in the crystallographically defined halogen-binding site. This study found that the best signal-to-noise ratio in cellular imaging is obtained with YFP variants that remain highly fluorescent and exhibit chloride affinity that matches the [Cl]_i_ at rest. At the end of their remarkable effort, the authors selected the double mutation Q69T-V163A, which possess a high affinity for chloride with a *K_d_* of 21.2 ± 2.2 mM at pH = 7.1. Unfortunately, Q69T-V163A remains highly dependent on pH changes with a *pK_a_* value of 6.4 ± 0.1. This new variant was substituted in the original Clomeleon to obtain SuperClomeleon (Grimley et al., 2013), in which three further optimizing modifications were introduced: (1) a brighter donor with the upgrade of CFP to Cerulean, (2) a shorter linker that optimizes the SNR of the biosensor in cultured neurons, and (3) the additional mutation S30R to enhance stability and brightness of both Cerulean donor and YFP-Q69T-V163A acceptor. Overall, SuperClomeleon exhibits a more than fourfold improvement in SNR over the original Clomeleon.

## CHLORIDE BINDING FLUORESCENT PROTEINS AND THEIR pH DEPENDENCE

Many of the listed chloride biosensors e.g., Clomeleon, Cl-Sensor and SuperClomeleon exhibit relatively high sensitivity to pH. Indeed, all available chloride biosensors, either ratiometric or non-ratiometric, are based on fluorescent proteins derived from the *Aequorea victoria* GFP. The common element is the T203Y mutation, which is present in all YFP and is required for chloride binding in GFP (Arosio et al., 2007). Unavoidably, fluorescence of all these YFP and GFP variants is pH-sensitive with acidification suppressing fluorescence emission and confounding the measurements of chloride concentration.

At low [Cl]_i_ (below 25 mM) the errors introduced by changes in intracellular pH values should be moderate, likely in the range from 10 to 20%. For instance, by loading the pH indicator BCECF-AM in YFP-H148Q-I152L expressing cells, variations of the fluorescence of YFP of about 15% were quantified for pH changes of 0.2 units around the physiological pH of 7.0 (Rhoden et al., 2007). Nevertheless in the same study, the pH-dependence of the dissociation constant remains the main source of error with reported iodide *K_d_* of 1.4, 2.0 and 2.4 mM at pH values of 6.8, 7.0 and 7.2, respectively. In agreement to Grynkiewicz equation (Grynkiewicz et al., 1985), measured ion concentration is directly proportional to *K_d_*; so an undetected pH change from 7.0 to 6.8 would lead to an overestimation of [Cl]_i_ of about 45%. These arguments suggest that the pH dependency of chloride biosensors is especially troublesome when shifts in intracellular pH are expected, e.g., during intense synaptic activity or epileptiform activity (Raimondo et al., 2012).

Measurement of the intracellular pH is thus needed to quantify the chloride affinity of YFP-based probes and ultimately to measure the exact [Cl]_i_ value. This disadvantage of GFP based sensors also offers a great opportunity: if properly designed, a GFP based chloride sensor would allow the simultaneous measurement of intracellular pH and chloride concentration. A response to this problem is provided by ClopHensor which was engineered to allow the simultaneous readout of pH and chloride (Arosio et al., 2010). Similar to the members of the Clomeleon family, ClopHensor is also a fusion construct linking two FP, DsRed-monomer and the EGFP-T203Y mutant, by a flexible 20-amino acid linker. EGFP-T203Y, also known as E^2^GFP, is a highly accurate ratiometric pH biosensor – with a *pK_a_* of about 6.8 at 37°C – with an isosbestic point in the excitation spectrum at 458 nm (Bizzarri et al., 2006). In EGFP, the single mutation T203Y introduces a specific halogen-binding site, which is located in a different position with respect to YFP-H148Q as shown by crystallographic analyses (pdb codes 1f09 and 2o2b). E^2^GFP loses its fluorescence upon chloride binding because of static quenching. This fact allows accurate pH measurements free from the influence of [Cl]_i_ variations, because the excitation and emission spectra remain unchanged through variations in chloride concentration. In the case of E^2^GFP, the chloride *K_d_* dependence on pH was thoroughly examined (Arosio et al., 2007) revealing cooperative binding of chloride and protons that can be mathematically expressed as follows:

(2) $$K_{d}=K_{d}^{1}\,\frac{1+10^{p\,K_{a}-p\,H}}{10^{p\,K_{a}-p\,H}}$$

where *K_d_^1^* is the chloride *K_d_* the proton-ligated form of E^2^GFP, and *pK_a_* is the logarithm of the association constant of proton to chloride-free E^2^GFP. DsRed-monomer fluorescence is insensitive to variation in either [Cl]_i_ or pH and can be excited and detected in a separate region of the spectral range relative to E^2^GFP. The red signal is used to compensate for changes in optical thickness, and provides an accurate reference signal to compute chloride concentration. Overall, ClopHensor spectral changes are ideally suited for ratiometric operation at three excitation wavelengths: 488, 458, and 560 nm. [Cl]_i_ measurements and chloride-independent pH measurements are derived from the ratios 458/560 and 488/458, respectively. In comparison to FRET based sensors, like Clomeleon, ClopHensor prevents the occurrence of subtle variations in the degree of FRET generated by pH-dependent variations of the GFP fluorescence. The cost of this is somewhat reduced time resolution, imposed by the need for sequential acquisition of three excitation wavelengths, and a less simple analysis. Chloride estimates in a region of interest must follow pH estimates and a corresponding calculation of the chloride *K_d_* (according to Eq. 2).

It has been recently reported that the expression of ClopHensor in hippocampal pyramidal neurons often results in highly heterogeneous DsRed intracellular aggregates. Substitution of tandem-Tomato for DsRed in ClopHensor yielded an improved version for neuronal imaging, **ClopHensorN**; although at the cost of a 50% size-increase (Raimondo et al., 2013). ClopHensorN permitted measurements of activity-dependent ion dynamics in hippocampal neurons.

## CALIBRATION

The sensor calibration is one of the most critical aspects for measuring absolute changes in [Cl]_i_. The precision and accuracy of measurements of [Cl]_i_ are determined by the parameters set during calibration. Calibration parameters can be divided into two groups, either those that are dependent on the optical setup or those that are dependent on the indicator chloride binding thermodynamic. Setup-dependent parameters account for different optical filters, detector gains, excitation intensity and general optical efficiency of the imaging setup. The experimenter should also be aware that the illuminated field is never uniform and generally dependent on excitation wavelength with both wide-field and laser-scanning microscopes. Indicator-dependent parameters should be absolute intrinsic properties of the molecular indicator accounting for the thermodynamics of chloride binding, namely chloride *K_d_* and its dependence on pH. In cell culture, intracellular – *in situ* – calibration of chloride indicators is generally performed using the ionophores nigericin and trybutyltin in buffer containing high concentration of potassium (from 100 to 150 mM) and a titrated concentration of chloride (Krapf et al., 1988). The addition of some other ionophores has been reported; e.g., monensin and ionomycin for clamping intracellular milieu at fixed values of sodium concentration (Harootunian et al., 1989) and the protonophore CCCP for controlling pH (Kneen et al., 1998). Alternative methods for *in situ* calibration in cell cultures were proposed by permeabilizing the cell membrane with digitonin (Kneen et al., 1998) or natural surfactants like the triterpenoid saponin, β-escin (Black et al., 2004; Nausch et al., 2008; Waseem et al., 2010). Although calibration procedures using β-escin and the ionophores nigericin and trybutyltin have been reported to yield encouragingly equivalent results (Watts et al., 2012), it is likely that the many variables potentially affecting *in situ* calibration will produce less precise estimates of the intrinsic molecular parameters when compared to spectroscopy measurements performed in a cuvette. GFP-based biosensors can be abundantly purified as recombinant proteins and the biosensor intrinsic parameters can be measured with high accuracy in a spectrofluorometer, under the same temperature, pH and concentration conditions of the final microscopy specimen. The same experiment with the purified biosensor can also be replicated using the microscope to confirm both setup-dependent and intrinsic biosensor parameters. For instance, ClopHensor is intrinsically characterized by two molecular parameters, the *pK_a_* and the chloride K_d_^1^ of the fully protonated form (see Eq. 2). In the original work these two parameters were determined, using the recombinant construct in cuvette, to be *pK_a_* = 6.81 ± 0.05 and K_d_^1^ = 13.1 ± 0.5 mM, and validated by *in situ* calibration on cell lines with reported *pK_a_* ranging from 6.78 to 6.84 and a chloride K_d_^1^ estimate of 14.4 ± 2.0 mM (Arosio et al., 2010). The good agreement between the calibration *in situ* and in cuvette suggests small effects of ionic strength and molecular crowding on the intrinsic parameters of ClopHensor. Yet, significantly different calibration parameters were reported for *in situ* calibrations of ClopHensor (**Table 2**), a difference that may lie in the calibration procedures.

**Table 2:** Properties of ratiometric indicators.

Biosensor name and main reference	Chloride *K_d_* (mM)	Ratiometric	Excitation (nm)	Emission (nm)	pKa
Clomeleon	167 ^*in vitro*^	Emission	440	485 (15)/530 (15)	5.2
(Kuner and Augustine, 2000)	137 ^*in vitro*Berglund et al. (2006)^		
	87 ^in cultured hippocampal neurons^		
Cl-sensor	28 ± 5	Excitation	440/480	535 (30)	7.1 ^at 4 mM KCl^
(Markova et al., 2008)	48.9 ^in cultured spinal neurons Waseem et al. (2010)^		
SuperClomeleon	8.1 ± 0.5	Emission	440	485 (15)/530 (15)	6.4 ± 0.1
(Grimley et al., 2013)	21.2 ± 2.2 ^*in vitro*^		
ClopHensor	49 ± 5*	Excitation	488/458	525 (25)	6.78 ± 0.04 ^in HEK 293 cells^
(Arosio et al., 2010)		458/561	640 (60)	
ClopHensor-H148G	21.4 ± 4.8 ^in CHO\ cells^		488/458	535 (15)	7.3 ^in CHO cells^
(Mukhtarov et al., 2013)		458/545	632 (30)	
ClopHensorN	39 ± 27	Excitation	488/458	525 (25)	7.45 ± 0.16 ^in hippocampal slices^
(Raimondo et al., 2013)		458/594	675 (25)	

*Propagating the error for the reported estimates of *k_d_* = 6.81 ± 0.05; *pK_a_* = 13.1 ± 0.5.

## FUTURE PERSPECTIVES AND OPEN ISSUES

We are entering the golden age of chloride imaging. New tools are being developed at an increasing rate and the technological efforts are encouraged by the understanding that the role of chloride homeostasis on the physiopathology of the brain is wider than expected. In this context, it is likely that the field will develop greatly and there are several issues that we can envision will be addressed. As our knowledge on the molecular determinants of chloride binding in GFP accumulates, we will be able to engineer ever improved biosensor variants that will optimally match [Cl]_i_. Many YFP variants were developed during the last decade (**Table 1**), while very few chloride binding GFP variants were reported (Arosio et al., 2010; Mukhtarov et al., 2013). We expect that new E^2^GFP variants will be developed, and it is likely that a chloride binding site will be inserted into other fluorescent proteins with different colors. The currently available ratiometric chloride biosensors are either based of the FRET between CFP and YFP variants or on the fluorescence emission ratio between GFP and RFP variants. New ratiometric design could be engineered to exploit alternative fluorescent proteins.

The substitution of the red fluorescence moiety in ClopHensor was demonstrated to improve substantially the ClopHensor potential in neuronal imaging and we envision substitution of the RFP with brighter and more photostable proteins in the future. The ideal RFP should be: a stable monomer not inclined to aggregate; pH independent with low *pK_a_*; and with high brightness, possibly even using 2-photon excitation. TagRFP-T (**Table 3**) appears to offer the potential for greater photostability (but not brightness) compared to existing ClopHensor-based indicators. Finally, another factor that can be considered in ratiometric biosensors based on the fusion between two FP is the photobleaching rate. If two FP are bleached at the same rate, their ratio would be unaffected.

**Table 3:** Alternative red fluorescent proteins and their thermodynamic and photo-physical properties.

FP	*pKa*	Excitation and emission (nm)	Relative brightness (%)	Relative photostability (%)	Selected reference
DsRed-monomer	4.5	556/586	10.3	9.2	Shaner et al. (2005)
mCherry	<4.5	587/610	47.0	55.2	Shaner et al. (2005)
tdTomato	4.7	554/581	279.4	56.3	Shaner et al. (2005)
mStrawberry	<4.5	574/596	76.5	8.6	Shaner et al. (2005)
mPlum	<4.5	590/649	12.1	30.5	Shaner et al. (2005)
TagRFP	3.1	555/584	142	21.3	Merzlyak et al. (2007), Day and Davidson (2009)
TagRFP-T	4.6		99	193.7	Shaner et al. (2008)
mKate2	5.4	588/633	74		Chudakov et al. (2010)
LSS-mKate2	2.7	453/605			Piatkevich et al. (2010)

*Photo-stability and brightness are reported as relative values with respect to EGFP.*

GABA_A_ activity leads to bicarbonate flux that, in principle, brings about a pH change inside the cell. As we have seen this tight connection between chloride and pH is also mirrored by the dual sensitivity of the fluorescent sensors. Presently, most of the available sensors do not allow the correction of chloride measurement for pH. The exception is ClopHensor, which allows the combined measurement of both ion species. In particular, it was recently demonstrated that ClopHensorN can be used to measure pH and chloride simultaneously in neurons (Raimondo et al., 2013). If one were interested only in chloride concentration, it might be a better choice to employ a sensor with pH independent fluorescence and pH independent chloride affinity. This would simplify the measurement and calibration processes, and ultimately should lead to a more accurate estimate of the absolute chloride concentration.

## *IN VIVO* IMAGING OF CHLORIDE CONCENTRATION IN ANESTHETIZED AND BEHAVING MICE

As occurred in the field of calcium imaging, the development of tools to measure chloride *in vivo*, where neural circuitry are fully preserved, will represent a dramatic improvement in our understanding of chloride regulation and of the mechanisms of GABA_A_ mediated inhibitory activity. This is especially relevant since many factors relative to slice preparation and maintenance might have profound effects on chloride regulation (Dzhala et al., 2012; Puskarjov et al., 2012). The availability of genetically encoded sensors will make it possible to target imaging to specific cell classes, and to perform longitudinal recordings on anesthetized and behaving mice. This is an especially relevant issue considering the potential effects that anesthesia might have on chloride concentration. Indeed, it is surprising that even when Clomeleon mice have been available for several years, there are no reports on chloride imaging *in vivo* yet. We can expect that the accurate measurement of chloride concentration *in vivo* would encounter some difficulties due to the sample thickness: to compute the chloride concentration with the available sensors it is necessary to measure the fluorescence emitted and/or excited at different wavelengths and to compare these results with calibration data obtained *in vitro*. Brain tissue is not transparent and both excitation and emission are attenuated in a wavelength dependent way. For example, at a depth of 150 μm, the photon flux is attenuated by a factor of 1.42 when the excitation is shifted from 950 nm to 800 nm, and the corresponding variation in fluorescence emitted greater than a factor of 2 (Brondi et al., 2012). Wavelength-dependent attenuation of excitation and emitted fluorescence does not affect our capacity of detecting relative changes of chloride *in vivo*, but it drastically affects the numerical accuracy of the estimate of absolute concentration. In spite of these problems, the importance for *in vivo* imaging of chloride homeostasis in the intact brain is obvious. Most measures of chloride dynamics have been performed during periods of abnormally large inhibitory activity, such as in the presence of epileptiform activity. *In vivo* imaging might reveal more subtle chloride fluxes that are activated in dendrites or in the soma by physiological activity. Up to now, an accurate mapping of intracellular chloride in cortical neurons has yet to be produced and it will be of obvious interest to image chloride compartmentalization within the dendritic tree and the cell body. Compartmentalization might arise by localized changes in homeostasis (Szabadics et al., 2006; Khirug et al., 2008), possibly linked to phosphorylation of KCC2 (Fiumelli et al., 2005; Kahle et al., 2013) and this might drastically affect the efficacy of inhibitory inputs placed in different neuronal compartments. Finally, sensory cortices offer the opportunity to study incoming GABAergic activity in response to physiological stimuli in regions where the functional architecture is well known.

Chloride imaging is playing a growing role in the understanding of brain diseases. Epilepsy is the most obvious pathology that involves an alteration in GABAergic transmission and, in fact, several studies have begun to dissect chloride regulation during seizures (Dzhala et al., 2005, 2010; Glykys et al., 2009; Lillis et al., 2012; Kaila et al., 2014). All these studies have been performed in slices, and hopefully, the next wave of studies will be performed on preparations that allow a better preservation of circuitry, perhaps by utilizing *in vivo* imaging of the hippocampus (see, for example, Mizrahi et al., 2004; Navarrete et al., 2012). While epilepsy and chloride homeostasis is certainly a very important field, it is becoming clear that the involvement of chloride regulation in brain physiopathology is far more extensive. The spectra of brain pathologies associated with defective inhibition is very wide including both acute situations such as ischemia or traumatic injury (Pond et al., 2006; Dzhala et al., 2012) and conditions such as fragile X and autism, (He et al., 2014; Tyzio et al., 2014) reviewed in (Pizzarelli and Cherubini, 2011; Deidda et al., 2014). It can be argued that dysregulation of chloride homeostasis might play an important part in any pathology associated to a defective equilibrium between excitation and inhibition. The availability of diuretics that alter the activity of chloride cotransporters and have a long clinical track record makes the modulation of chloride homeostasis an attractive target for pharmacological intervention (Löscher et al., 2013; Deidda et al., 2014; Puskarjov et al., 2014). Since the genetically encoded biosensors can be efficiently transfected during late fetal life (dal Maschio et al., 2012), it will also be feasible to screen different murine models of brain pathologies for alterations in the development of chloride regulation. In summary, *in vivo* imaging will likely offer a powerful pre-translational tool for the direct testing of drugs acting on chloride homeostasis in different models of brain diseases. A luminous future for chloride imaging is about to begin.

## Conflict of Interest Statement

The authors declare that the research was conducted in the absence of any commercial or financial relationships that could be construed as a potential conflict of interest.
